# Effect of Indian Polyvalent Antivenom in the Prevention and Reversal of Local Myotoxicity Induced by Common Cobra (*Naja naja*) Venom from Sri Lanka In Vitro

**DOI:** 10.3390/toxins13050308

**Published:** 2021-04-26

**Authors:** Umesha Madhushani, Prabhani Thakshila, Wayne C. Hodgson, Geoffrey K. Isbister, Anjana Silva

**Affiliations:** 1 Department of Parasitology, Faculty of Medicine and Allied Sciences, Rajarata University of Sri Lanka, Saliyapura 50008, Sri Lanka; madhushanih.h.u@gmail.com (U.M.); prabhanithakshila123@gmail.com (P.T.); 2 Monash Venom Group, Department of Pharmacology, Biomedical Discovery Institute, Monash University, Clayton 3800, Australia; wayne.hodgson@monash.edu; 3 Clinical Toxicology Research Group, University of Newcastle, Callaghan 2308, Australia; geoff.isbister@gmail.com

**Keywords:** myotoxicity, local necrosis, antivenom, cobra bite, envenoming, reversal

## Abstract

Bites by many Asiatic and African cobras (Genus: *Naja*) cause severe local dermonecrosis and myonecrosis, resulting in permanent disabilities. We studied the time scale in which two Indian polyvalent antivenoms, VINS and Bharat, remain capable of preventing or reversing in vitro myotoxicity induced by common cobra (*Naja naja*) venom from Sri Lanka using the chick biventer cervicis nerve-muscle preparation. VINS fully prevented while Bharat partially prevented (both in manufacturer recommended concentrations) the myotoxicity induced by *Naja naja* venom (10 µg/mL) when added to the organ baths before the venom. However, both antivenoms were unable to reverse the myotoxicity when added to organ baths 5 and 20 min post-venom. In contrast, physical removal of the venom from the organ baths by washing the preparation 5 and 20 min after the venom resulted in full and partial prevention of the myotoxicity, respectively, indicating the lag period for irreversible cellular injury. This suggests that, although the antivenoms contain antibodies against cytotoxins of the Sri Lankan *Naja naja* venom, they are either unable to reach the target sites as efficiently as the cytotoxins, unable to bind efficiently with the toxins at the target sites, or the binding with the toxins simply fails to prevent the toxin-target interactions.

## 1. Introduction

Snake envenoming is a significant public health issue in the tropics [1]. Literature-based estimates suggest 0.4–1.8 million envenomings, and 20,000–94,000 deaths occur each year due to snakebites globally [2]. South Asia records the highest snakebite burden in the world, with over two-thirds of the snakebite mortality [2,3]. Although most life-threatening effects of snake envenoming are acute, a large number of victims survive with permanent disabilities due to the local necrotic effects of the venom [4].

Many African and some Asiatic cobras (genus *Naja*) are known to cause severe local necrosis in envenomed patients [5,6]. The tissue-necrotizing effect of cobra venoms is primarily due to the cytotoxic three-finger toxins (cardiotoxins) of their venoms, which are polypeptides with 59–62 amino acid residues and four disulfide crosslinks shaping into three protruding finger-like structures from a hydrophobic core [7,8]. Cytotoxic three-finger toxins result in pore formation on cell membranes and disruption of cell membrane integrity, initiating cell death [9,10]. Some cytotoxic three-finger toxins in elapid venoms, including cobra venoms, act synergistically with the venom phospholipases A_2_ [11].

The common (Indian) cobra (*Naja naja*) is distributed throughout the Indian subcontinent, and is one of the four major venomous snakes (big four) responsible for a large number of envenomings in the region [3,6,12,13]. Envenoming by the common cobra commonly results in life-threatening neuromuscular paralysis and severe local dermonecrosis and myonecrosis, sometimes requiring prompt surgical interventions, such as amputations [12,14]. In agreement with the clinical profile of envenomed patients, a venom study by Sintiprungrat et al., showed relative abundances of cardiotoxins as high as 71.5 and 69.3% in Sri Lankan and Indian common cobra venoms, respectively [15]. The same study has shown the presence of cytotoxic three-finger toxins unique to common cobra venom from Sri Lanka and India, suggesting geographical differences between the venoms.

Antivenoms have been used to treat snake envenoming for more than a century. However, the effectiveness of antivenom therapy in preventing or reducing the local tissue damage in snake envenoming is not well-supported by current evidence [12,16,17]. In addition, different clinical reports provide contrasting observations on the effectiveness of early administration of antivenom in preventing or controlling local necrosis in envenoming by cobra species such as the Chinese cobra (*Naja atra*) [17,18]. Indian polyvalent antivenom is raised against the venoms of the “big four snakes” of the Indian subcontinent, i.e., Russell’s viper, saw-scaled viper, Indian krait, and Indian cobra. Poor effectiveness of Indian polyvalent antivenom in preventing or treating local necrosis caused by Indian cobra bites in Sri Lanka has previously been reported in observational studies [12]. The efficacy of Indian polyvalent antivenoms in neutralizing the lethal and neurotoxic effects of Indian cobra venom from Sri Lanka has previously been tested using experimental studies [19,20]. However, functional studies focusing on testing the efficacy of the Indian polyvalent antivenom in neutralizing local cellular damage caused by the Indian cobra venom are unavailable. The time scale within which the Indian polyvalent antivenom remains effective in preventing/reversing the local tissue damage induced by Indian cobra venom has not been previously explored.

This study aims to explore the time scale in which the two commonly used Indian polyvalent antivenom brands are effective in preventing and arresting/reversing the local myotoxicity induced by Indian cobra venom from Sri Lanka.

## 2. Results

### 2.1. In Vitro Muscle Injury Caused by Naja naja Venom

**Figure 1:**
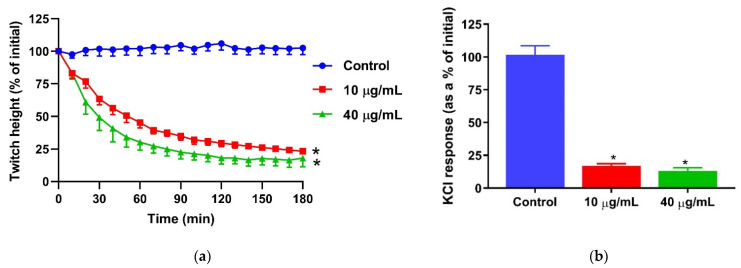
In vitro myotoxicity of *Naja naja* venom on the chick biventer nerve/muscle preparation: (**a**) inhibition of direct twitches by the venom (* significantly different from the control at 180 min; *p* < 0.05; one-way ANOVA followed by Tukey’s post hoc test, *n* = 5–7); (**b**) inhibition of contractile responses to KCl (40 mM) by the venom (* significantly different from the control at 180 min; *p* < 0.0001; one-way ANOVA followed by Tukey’s post hoc test, *n* = 5–7).

Common cobra venom (10 and 40 µg/mL) caused inhibition of direct twitches in the chick biventer nerve muscle preparation (Figure 1a) and abolished the response to KCl (40 mM; Figure 1b), indicative of skeletal muscle damage caused by the venom.

### 2.2. Prevention of the Naja naja Venom-Mediated In Vitro Muscle Injury by Indian Polyvalent Antivenoms

**Figure 2:**
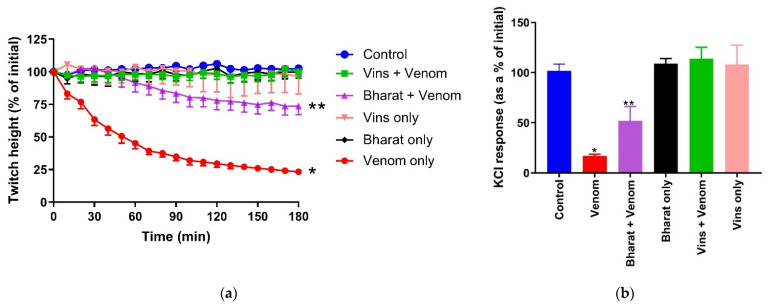
Prevention of the in vitro myotoxicity of *Naja naja* venom (10 µg/mL) by manufacturer-recommended quantities of VINS and Bharat antivenoms in the chick biventer nerve/muscle preparation: (**a**) prevention of the venom-mediated inhibition of direct twitches by the two antivenoms (* significantly different from the control at 180 min; *p* < 0.0001; ** significantly different from both the control and venom at 180 min; *p* < 0.05; one-way ANOVA followed by Tukey’s post hoc test, *n* = 3–7); (**b**) the effect of the antivenoms on the venom-mediated inhibition response to KCl (40 mM; * significantly different from the control; ** significantly different from both the control and venom; *p* < 0.05; one-way ANOVA followed by Tukey’s post hoc test, *n* = 4–7).

Of the two concentrations of venom, 10 µg/mL was selected for antivenom and washing experiments in order to minimize the concentration of antivenom in the bath so that it would not alter the physiological conditions in the bath. Manufacturer-recommended quantities of VINS and Bharat antivenoms fully or partially, respectively, prevented *Naja naja* venom (10 µg/mL)-mediated inhibition of direct twitches in the chick biventer nerve/muscle preparation (Figure 2a). Similarly, VINS antivenom fully prevented the inhibition of responses to KCl, while Bharat antivenom partially prevented the inhibition of responses to KCl (Figure 2b). The two antivenoms alone had no significant effect on the tissues.

### 2.3. Reversibility of Naja naja Venom-Mediated In Vitro Muscle Injury by Indian Polyvalent Antivenoms

**Figure 3:**
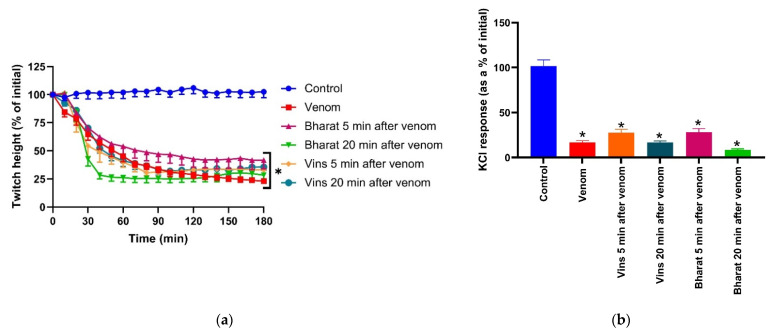
Reversibility of the *Naja naja* venom-induced myotoxicity in the chick biventer nerve/muscle preparation by VINS and Bharat antivenoms post-venom: (**a**) reversibility of the inhibition of direct twitches when the antivenoms were added 5 min or 20 min after the venom (* significantly different from the control at 180 min; *p* < 0.0001; one-way ANOVA followed by Tukey’s post hoc test, *n* = 4–7); (**b**) the effect of the antivenoms 5 min or 20 min post-venom on the venom-mediated inhibition of the response to KCl (40 mM; * significantly different from the control; *p* < 0.0001; one-way ANOVA followed by Tukey’s post hoc test, *n* = 4–7).

*Naja naja* venom (10 µg/mL)-mediated inhibition of direct twitches and inhibition of the response of the muscle to KCl (40 mM) of chick biventer nerve/muscle preparation was not reversed by either VINS or Bharat antivenoms when added 5 or 20 min post-venom (Figure 3a,b).

### 2.4. Reversibility of Naja naja Venom-Mediated In Vitro Muscle Injury by Washing the Preparation

**Figure 4:**
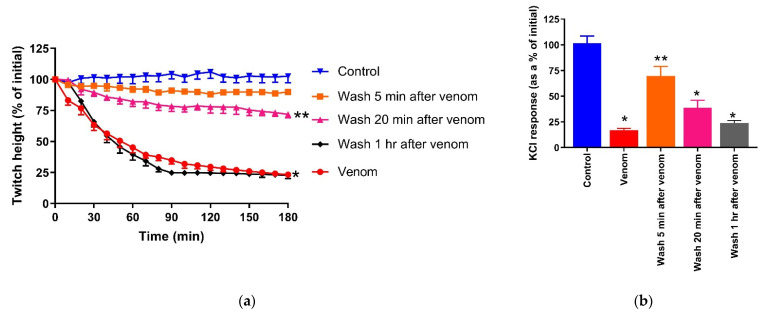
Reversibility of *Naja naja* venom-induced myotoxicity in the chick biventer nerve/muscle preparation by washing the preparation post-venom: (**a**) reversibility of the inhibition of direct twitches (* significantly different from the control at 180 min, ** significantly different from both the control and venom; *p* < 0.05; one-way ANOVA followed by Tukey’s post hoc test, *n* = 4–7); (**b**) the effect of washing post-venom on the inhibition of responses to KCl (40mM; * significantly different from the control, ** significantly different from both the control and venom; *p* < 0.05; one-way ANOVA followed by Tukey’s post hoc test, *n* = 4–7).

*Naja naja* venom (10 µg/mL)-mediated inhibition of direct twitches of the chick biventer nerve/muscle preparation was fully reversed by physical removal of the venom from the organ bath by washing the preparation 5 min post-venom. However, washing after 20 min only partially reversed the inhibitory effects, while washing after 1 h did not reverse the inhibition of direct twitches (Figure 4a). Washing the preparation after 5 min partially reversed the abolition of the response to KCl, and washing after 20 min or 1 h did not reverse the abolition of the response to KCl (Figure 4b).

## 3. Discussion

This study investigated the in vitro efficacy of two commercial Indian polyvalent antivenoms on *Naja naja* venom-mediated muscle damage in a skeletal muscle preparation. When added prior to the venom in manufacturer-recommended concentrations, VINS antivenom fully prevented myotoxicity, while Bharat antivenom partially prevented myotoxicity. However, both antivenoms were unable to reverse the venom-mediated muscle damage when added to baths after the venom. Interestingly, physical removal of the venom from the bath by washing the muscle preparation 5 min and 20 min after venom, fully and partially, prevented the myotoxic effect of the venom, respectively.

Previous clinical studies have shown the ineffectiveness of the Indian polyvalent antivenoms against the local effects of *Naja naja* envenoming, evident from the lack of correlation of the severity of the local envenoming to the bite-to-antivenom time gap [12,21]. In this study, we demonstrated the ability of the two antivenoms to prevent the *Naja naja* venom-induced muscle injury (when added to the bath before the venom), which indicated the presence of specific antibody fragments against cytotoxic components of the Sri Lankan *Naja naja* venom. This is in agreement with the previous venom-antivenom binding studies, in vitro neurotoxicity studies, and rodent lethality studies. These studies demonstrated the successful binding of both VINS and Bharat antivenoms with the Sri Lankan *Naja naja* venom antigens based on the prevention of in vitro and in vivo neurotoxic and lethal effects of the venom when antivenom and venom were pre-incubated or pre-mixed [19]. However, the clinical effectiveness of an antivenom depends on the ability of the antibody fragments not only to bind with the toxins, but to access the toxins already at the target sites, so that they can bind to them. Once antivenom binds it can then prevent the toxins from interacting with their target and, possibly, reverse the pathophysiological processes that have already begun [16]. Neutralization of toxins is only achieved if the antibodies or their fragments interfere directly with the toxin-target/substrate interaction. In addition to the ability to neutralize toxin activity, preventing the toxin from reaching the target via different mechanisms, such as steric hindrance, trapping the toxin in the central compartment, and enhancing the elimination of the toxin, is also important for antivenom efficacy [16,22,23].

In the prevention experiments, the addition of the antivenom to the bath before the addition of the venom allows antibody fragments time to bind and trap the venom cytotoxins, preventing them from reaching the target muscle cell membranes. However, in reversal experiments, the prior addition of the venom to the organ bath allows the cytotoxins to reach the target muscle cell membranes and therefore allows insufficient time for the antivenom to bind. In contrast, physical removal of the toxins by washing the preparation 5 min and 20 min after addition of the venom fully and partially prevented the cytotoxic action, respectively. This indicates that there is a lag period required for the cytotoxins to start irreversible cellular injury. However, the failure of both Indian polyvalent antivenoms to prevent *Naja naja* venom-mediated muscle injury even after 5 min of exposure to venom suggests that (a) the F(ab’)2 antibody fragments (~110 kDa) are unable to reach the target sites as quickly as most of the cytotoxins do (<8 kDa), which are much smaller in size, (b) the binding of the antibody fragments to the antigenic sites of the toxins already in target sites fails to prevent the toxin-target interactions [7,8,16,22], or (c) the time for antivenom to bind to venom is longer than the short delay before the toxins cause irreversible injury.

In this study, we used chick biventer nerve/muscle preparation to study the effect of antivenom on the cytotoxic effects of *Naja naja* venom. The World Health Organization recommends conventional antivenom standardization tests, such as the rodent skin necrotic patch model (minimum necrotizing dose median effective dose), require venom to be pre-incubated with the antivenom before the venom/antivenom mix is applied to the rodent tissues [24]. This approach does not mirror the real-life situation, in which there is a delay between envenoming and the administration of antivenom. The rodent skin necrotic patch model requires experimental animals to be kept alive for 72 h post-envenoming, allowing time for the generation of a necrotic patch on the skin. Such methods result in the suffering of the test animal. An in vivo rodent myotoxicity model, in which venom is injected intramuscularly into the thigh muscles of mice and the serum or plasma creatine kinase activity after 3 h is measured to quantify the muscle injury, has been previously described [25]. Although the method may accommodate a time gap between venom injection and antivenom administration, mirroring a real-life situation, the utility of creatine kinase as a marker of venom-induced muscle damage before 6 h has previously been questioned based on the observed lag period between venom pharmacokinetics and the rise of creatine kinase by other studies [26]. Furthermore, both the skin necrotic patch model and in vivo myotoxicity model are less useful to test venoms with significant quantities of neurotoxins, which are likely to kill the experimental animal before the necrotic effects or the creatine kinase rise can be observed.

We used an in vitro skeletal muscle preparation. Although the chick biventer myotoxicity model is not an alternative to the skin necrotic patch model in assessing dermonecrosis, the model allowed us to quantify the muscle damage induced by direct contact with venom, as well as examine the effects of antivenom after a delay following venom administration. This better mirrors real-life envenoming. Furthermore, the contrasting results between the antivenom prevention (i.e., antivenom before venom) and reversal (i.e., antivenom after venom) experiments observed in our study, as well as our previous studies on neurotoxic venoms, highlight the limitations of applying conventional antivenom efficacy testing to explore the limited clinical effectiveness of antivenoms, in which there is a delay between envenoming and the administration of antivenom [27]. This further highlights the appropriateness of antivenom reversal experiments using in vitro chick biventer preparation in exploring the role of antivenom in treating the local effects induced by snake venoms.

## 4. Materials and Methods

### 4.1. Venom

Desiccated and crystallized common cobra (*Naja naja*) venom from Sri Lanka was used for this study. The venom was dissolved in MilliQ water and stored at −80 °C until required. Protein quantification of the venom was carried out using a BCA Protein Assay Kit (Thermo Fisher Scientific, Rockford, IL, USA) per manufacturer’s instructions.

### 4.2. Antivenom

Indian polyvalent antivenoms manufactured by VINS Bioproducts Limited (Telangana, India; Batch No: 01AS17008; date of expiry: 03.2021) and Bharat Serums and Vaccines Limited (Maharashtra, India; Batch No: A05317042; date of expiry: 01.2021) were used for this study. The antivenom was reconstituted in 2 mL of distilled water instead of the 10 mL recommended by the manufacturer to minimize the final volume of the antivenom solution to be added into the organ baths. According to the manufacturer, 1/10 of the content of a single antivenom vial (0.2 mL of the antivenom solution made for the present study) neutralizes 0.6 mg of Indian cobra venom. In all experiments, the required antivenom amount to neutralize the venom amount in the organ bath was calculated based on the above recommendation by the manufacturer.

### 4.3. Chick Biventer Cervicis Nerve-Muscle Preparation

Male chickens (aged 4–10 days) were humanely killed by exsanguination following CO_2_ inhalation. Biventer cervicis nerve-muscle preparations were dissected out and then mounted on wire tissue holders under 1 g resting tension in 25 mL organ baths. The tissues were maintained at 34 °C and bubbled with 95% O_2_ and 5% CO_2_ in physiological salt solution of the following composition (mM); 118.4 of NaCl, 4.7 of KCl, 1.2 of MgSO_4_, 1.2 of KH_2_PO_4_, 2.5 of CaCl_2_, 25 of NaHCO_3_ and 11.1 of glucose. Direct twitches were evoked by stimulating the muscle belly (rate: 0.1 Hz; pulse duration: 2 ms) at a supramaximal voltage (20–30 V) with the two electrode loops directly encircling the muscle belly, using an electronic stimulator (ADInstruments Pty Ltd., Bella Vista, NSW, Australia). Contractile responses of the tissues to KCl (40 mM for 30 s) was obtained in the absence of muscle stimulation. Following that, direct stimulation of the muscle was resumed. The preparations were stimulated for 10 min before the addition of venom or control (physiological salt solution). In antivenom prevention experiments, antivenom was added to the bath after 10 min of direct stimulation of the tissues, and the venom was added once the tissues were equilibrated in antivenom for 10 min. After adding venom, washing or the addition of antivenom was practiced at different time intervals. All experiments were run for 3 h. At the conclusion of the experiment, KCl was re-added as above.

### 4.4. Data Analysis and Statistics

Direct twitch responses and responses to KCl were measured via a MLT0201 force transducer (ADInstruments Pty Ltd., Bella Vista, NSW, Australia) and recorded on a PowerLab system (ADInstruments Pty Ltd., Bella Vista, NSW, Australia). Responses were expressed as the percentages of their pre-venom values. To compare the responses to exogenous agonists following the administration of venom, a one-way ANOVA was used. All ANOVAs were followed by Tukey’s multiple comparison post-tests. Data are presented in the form of the mean +/− standard error of the mean (SEM) of three to seven experiments. All statistical analyses and the presentation of data were generated using GraphPad Prism 9.0.1 software (GraphPad Software Inc., La Jolla, CA, USA). For all statistical tests, *p* < 0.05 was considered statistically significant.

### 4.5. Animal Ethics

All animal experiments used in this study were approved by the Ethics Review Committee of the Faculty of Medicine and Allied Sciences, Rajarata University of Sri Lanka, date of approval: 1 July 2018 (Approval no: ERC/2018/13).

## Data Availability

Data is contained within the article.
